# Performance Optimization of a Condenser in Ocean Thermal Energy Conversion (OTEC) System Based on Constructal Theory and a Multi-Objective Genetic Algorithm

**DOI:** 10.3390/e22060641

**Published:** 2020-06-09

**Authors:** Zhixiang Wu, Huijun Feng, Lingen Chen, Yanlin Ge

**Affiliations:** 1 Institute of Thermal Science and Power Engineering, Wuhan Institute of Technology, Wuhan 430205, China; zhixiangwuhg@outlook.com (Z.W.); huijunfeng@139.com (H.F.); geyali9@hotmail.com (Y.G.); 2 School of Mechanical and Electrical Engineering, Wuhan Institute of Technology, Wuhan 430205, China; 3 College of Power Engineering, Naval University of Engineering, Wuhan 430033, China

**Keywords:** plate condenser, ocean thermal energy conversion system, constructal theory, multi-objective optimization, generalized thermodynamic optimization, multi-objective genetic algorithm

## Abstract

Constructal optimization of a plate condenser with fixed heat transfer rate and effective volume in ocean thermal energy conversion (OTEC) system is performed based on constructal theory. Optimizations of entropy generation rate (${\dot{S}}_{g}$) in heat transfer process and total pumping power ($P_{\mathrm{sum}}$) due to friction loss are two conflicting objectives for a plate condenser. With the conventional optimization method, the plate condenser is designed by taking a composite function (CF) considering both ${\dot{S}}_{g}$ and $P_{\mathrm{sum}}$ as optimization objectives, and employing effective length, width, and effective number of heat transfer plates as design variables. Effects of structural parameters of the plate condenser and weighting coefficient of CF on design results are investigated. With a multi-objective genetic algorithm, the plate condenser is designed by simultaneously optimizing ${\dot{S}}_{g}$ and $P_{\mathrm{sum}}$, and the Pareto optimal set is obtained. The results demonstrate that CFs after primary and twice-constructal optimizations are respectively reduced by 7.8% and 9.9% compared with the initial CF, and the effective volume of the plate condenser has a positive impact on the twice minimum CF. Furthermore, the Pareto optimal set can provide better selections for performance optimizations of plate condensers.

## 1. Introduction

With the increasing demand for energy, it is urgent to find new energy. Ocean thermal energy which has the characteristics of a large reserve, renewability, and sustainability has been receiving wide attention. The ocean thermal energy conversion (OTEC) system was proposed by D’Arsonval [1], then many scholars performed plenty of research on it [2,3,4,5,6,7,8,9]. The closed OTEC system outputs energy (power and electricity) by utilizing the low boiling point refrigerant to exchange heats with the warm and cold seawaters to form the thermodynamic cycle. Therefore, the heat exchanger (HE) is an indispensable component of the OTEC system.

The condenser is one of the HEs in the OTEC system, and it usually adopts the plate HE [10,11,12,13,14,15,16] due to the constraints of the limited space volume and the small temperature difference between the warm and cold seawaters. It has been an important work to analyze the performance and optimize the structure for the plate condenser. By using the classical method, Nakaoka and Uehara [17] studied the heat transfer characteristic of a shell-and-plate-type condenser in an OTEC system, and proposed the empirical correlations to predict the heat transfer coefficients (HTCs) on the water side and condensation side. Yan et al. [18] researched the condensation pressure drop and HTC of a plate HE with refrigerant R134a, and analyzed the influences of refrigerant mass flux and vapor quality on the performance of the plate HE. Wang et al. [19] conducted an investigation on the flow characteristic for a plate HE under the conditions of partial condensation and complete condensation, and obtained the condensation pressure drop from the experimental data. Han et al. [20] proposed the formulas to calculate the friction factor and Nusselt number of the plate HE by considering the corrugation angle and corrugation wavelength. García-Cascales et al. [21] analyzed the calculation formulas of condensation HTCs in different plate HEs, and found that calculated results and experimental results are in good agreement. Longo et al. [22] found that the condensation HTC of the plate HE with refrigerant HFO1234ze (E) is less affected by saturation temperature while greatly affected by the refrigerant mass flux. Comparing the refrigerant HFO1234ze (E) with HFC134a, it was found that the HFC134a shows higher HTC and lower friction pressure drop. According to further experimental analyses and researches, Longo et al. [23] put forward a new formula to predict the condensation HTC of the plate HE with forced convection. Eldeeb et al. [24] and Shon et al. [25] compared the condensation HTCs of the plate HE with different working fluids, and found that ammonia shows higher heat transfer performance than other working fluids. Shon et al. [26] proposed the empirical correlations to predict the condensation pressure drop and HTC of the plate HE with refrigerant R-1233zd (E).

Constructal theory [27,28,29,30,31,32,33,34,35,36,37,38,39,40,41,42,43,44,45,46,47] was put forward by Professor Bejan in 1996. The corresponding constructal law [27,28] is “For a finite-size flow system to persist in time (to live), its configuration must change in time such that it provides easier and easier access to its currents.” The structural designs for living and non-living systems can be performed by applying constructal theory, including the structural designs of HEs. Vargas and Bejan [48] conducted an optimization for the aspect ratio of a crossflow HE, and analyzed the influences of smooth and finned heat transfer surfaces on the aspect ratio. It was found that the optimal constructs of the HE with two heat transfer surfaces are approximately the same. Bejan [49] constructed a multistage HE with the aim of the minimum pumping power and maximum heat exchange quantity, and found that the optimal spacings of the first-construct and elemental channels will lead the total pumping power to be minimum. Sotoodeh et al. [50] carried out a comparison between the ordinary single-scale plate-fin HE and constructal multi-scale plate-fin HE with the same volume and heat transfer area, and found that the constructal multi-scale plate-fin HE has higher heat recovery ability than the ordinary single-scale plate-fin HE. Xie et al. [51] optimized the pin-fins of the HE and obtained its optimal diameter, length, and shape. At the same time, it was found that the heat storage capacity of the HE after constructal optimization improves by 10.2%. Bejan et al. [52] obtained the optimal construct of a crossflow HE at the maximum heat transfer rate (HTR), and found that the optimal construct changes with the total flow-channel number and total volume of the HE. Besides the crossflow HE, Bejan et al. [53] also studied the HEs with different sizes, and found that the thermodynamic loss of flow structure can be reduced by simultaneously optimizing the shape and size of the HE. Feng et al. [54] conducted a constructal design for the disc-shaped HE, and obtained the optimal tube length at the maximum thermal efficiency. Feng et al. [55] and Cai et al. [56] investigated the performance characteristics of the shell-and-tube HEs with working fluids of R245fa and ammonia–water, respectively. And the results indicated the overall performances of the HEs after constructal designs were improved. Wu et al. [57] applied the plate HE to the evaporator of OTEC system, and obtained the optimal width of the heat transfer plate (HTP) by optimizing the total pumping power due to friction loss. Hajabdollahi [58] carried out multi-objective optimization for a plate-fin HE by simultaneously maximizing the thermal effectiveness and minimizing the annual cost, and discovered the optimal effectiveness of constructal plate-fin HE was higher than 0.747. Feng et al. [59] conducted an optimization for superheater in the supercharged boiler with the fixed total HE area, and found that the optimization objective was decreased by 2.6% after optimizing the tube outer diameter. Ariyo and Bello-Ochende [60] optimized the structural and flow parameters of a microchannel HE with the fixed total volume, and obtained the constructal microchannel HE with the minimum thermal resistance.

There are lots of studies about the performance analyses of the plate HE, but fewer optimizations in respect to the construct of the plate HE, especially in the case of the plate HE being applied to an OTEC system. However, the construct determines the performance of the plate HE, which has a crucial influence on an OTEC system. To enhance the comprehensive performance and find the optimal construct of the plate condenser, this paper will establish a composite function (CF) which considers both entropy generation rate (EGR) in heat transfer process and total pumping power due to friction loss as optimization objective, and apply constructal theory to optimize the HTP effective length, width, and effective number when the effective volume and total HTR of the plate condenser are fixed. Then the impacts of some parameters on the constructal optimization results (CORs) will be dissected. Furthermore, Pareto optimal set of the plate condenser based on the multi-objective genetic algorithm will be given by simultaneously minimizing the EGR in heat transfer process and the total pumping power due to friction loss. The obtained results can be applied to guide the designs for plate condensers in OTEC system.

## 2. Plate Condenser and Its Performance

**Figure 1:**
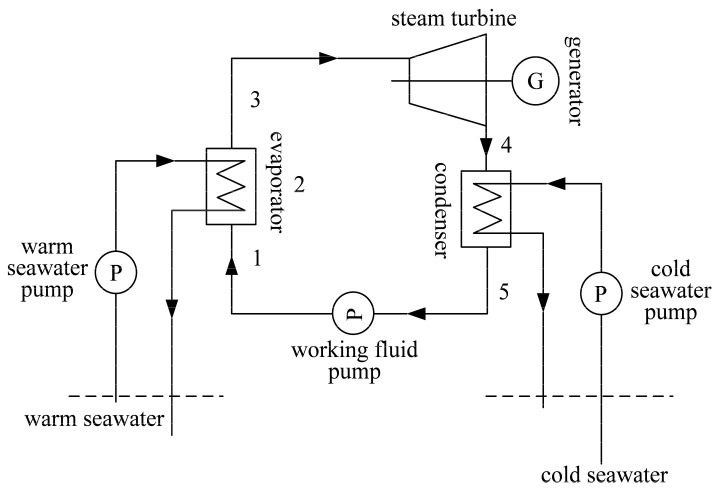
Schematic diagram of the closed ocean thermal energy conversion (OTEC) system.

**Figure 2:**
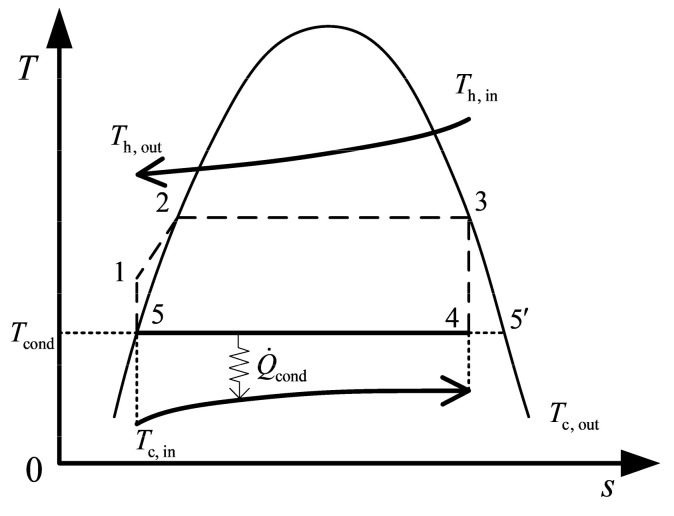
*T-s* diagram of the organic Rankine cycle in OTEC system.

Figure 1 shows a schematic diagram of the closed OTEC system, which takes the cold and warm seawaters as the heat sink and heat source, respectively. Because of the low temperatures of seawater, the OTEC system chooses the low boiling point substance as the working fluid. Based on References [13,15,16,61,62,63], ammonia is chosen as the working fluid for OTEC system after synthetically considering the environmental protection and thermodynamic performance. In Figure 2, the solid line 4→5 in the cycle *T-s* diagram represents the condensation process. The cold seawater absorbs heat from the working fluid, and its temperatures are $T_{c,\mathrm{in}}$ and $T_{c,\mathrm{out}}$ at the inlet and outlet of the plate condenser, respectively. The working fluid releases heat to the cold seawater and occurs a phase transition in the plate condenser. The condensation pressure and temperature of the working fluid are $p_{\mathrm{cond}}$ and $T_{\mathrm{cond}}$, respectively.

### 2.1. Structure of Plate Condenser

**Figure 3:**
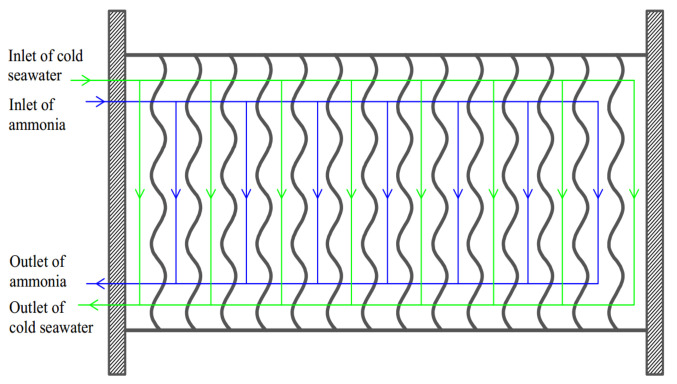
Flow schematic diagram of a plate heat exchanger (HE).

**Figure 4:**
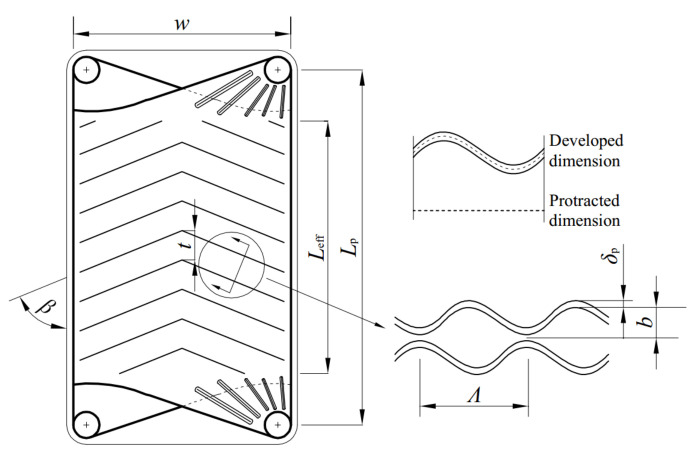
Structure diagram of a chevron plate HE.

The plate HE has the advantages of the high HTC, small area, large logarithmic mean temperature difference, and small terminal temperature difference, therefore the plate HE is applied to the condenser in OTEC system. Currently, the stainless steel HE is the most widely used in the plate HEs, but it is not resistant to corrosion. While the OTEC system needs the plate material to be corrosion resistant. Although titanium has strong corrosion resistance, its price is too expensive. Researchers have found that the service life of the brazed aluminum HE can reach more than 30 years under the high-temperature seawater corrosion condition. Thus, the brazed aluminum is chosen as the plate material in the OTEC system [10,64,65]. Figure 3 depicts the flow schematic diagram of a plate HE. Plate heat exchangers are usually counterflow when the heat sink and working fluid are both single phases. While when the working fluid undergoes phase transition in the plate condensers, its pressure is constant after ignoring the flow pressure drop. At this time, the pressure drop and discharge of the heat sink are considered more than the flooding of the working fluid. If the plate condensers are still counterflow, its bottom has much heat sink, which leads to the large temperature difference and large flow pressure drop. Moreover, the counterflow will reduce the condensation temperature and lower heat exchange efficiency. Thus, the flow channels of cold seawater and working fluid in plate condenser both adopt the down-flow and single flow path configuration [12] to reduce the pressure drop and facilitate the condensate discharge. The combined forms of flow channels for working fluid and cold seawater are configured as $\left(1\times N_{\mathrm{wf}}\right)/\left(1\times N_{c}\right)$, where $N_{\mathrm{wf}}$ and $N_{c}$ (equals to $N_{\mathrm{wf}}+1$) are the flow channel numbers of working fluid and cold seawater, respectively. Figure 4 depicts the structure diagram of a chevron plate HE, and the structural parameters $L_{p}$, $L_{\mathrm{eff}}$, w, β, Λ, t, b, and $\delta_{p}$ are the length, effective length, width, corrugation angle, corrugation wavelength, corrugation pitch, adjacent plate spacing, and thickness of the HTP, respectively. The relationship among β, Λ, and t is $t=\Lambda /\sin\left(\beta \right)$.

For the chevron plate HE, the hydraulic diameter ($d_{h}$) is:

(1) $$d_{h}=2b/\phi$$

where ϕ is the surface enlargement factor, i.e., the ratio of developed dimension to protracted dimension in Figure 4. When HTP corrugation section is sinusoidal, ϕ is approximately expressed as:

(2) $$\phi \approx \left(1/6\right)\left(1+\sqrt{X^{2}+1}+4\sqrt{X^{2}/2+1}\right)$$

where X (equals bπ/Λ) is the HTP dimensionless corrugation parameter.

The protracted area ($A_{\mathrm{pro}}$) of the single HTP is:

(3) $$A_{\mathrm{pro}}=L_{\mathrm{eff}}w$$

And the heat transfer area ($A_{0}$) (i.e., developed area) of the single HTP is:

(4) $$A_{0}=\phi A_{\mathrm{pro}}=\phi L_{\mathrm{eff}}w$$

The total heat transfer area ($A_{\mathrm{sum}}$) and effective volume of the plate condenser are:

(5) $$A_{\mathrm{sum}}=N_{\mathrm{eff}}A_{0}$$

(6) $$V_{\mathrm{eff}}=\left(N_{\mathrm{eff}}+1\right)L_{\mathrm{eff}}wb+N_{\mathrm{eff}}L_{\mathrm{eff}}w\delta_{p}$$

where $N_{\mathrm{eff}}$ (equals $2N_{\mathrm{wf}}$) is the HTP effective number.

### 2.2. Assumptions of Model

There are some assumptions to simplify the model and its calculations based on References [11,66,67]:

(1) The flow in the plate condenser is a stable state and homogeneous for flow direction.
(2) The working fluid is a two-phase state at the inlet of the plate condenser.
(3) Considering that the cold seawater is enough, the working fluid will be cooled to a saturated liquid state (SLS).
(4) The pressure drops at the manifolds and ports are ignored because only the HTP structure is studied in this paper. The influences of the manifolds and ports on the pressure drops and overall performance of OTEC system will be studied in future work.

### 2.3. Performance of Plate Condenser on Working Fluid Side

The vapor quality (x) of the working fluid changes continuously in the condensation process, and it has a significant impact on the performance of the plate condenser. Therefore, calculating the performance of the plate condenser by applying the average vapor quality ($x_{\mathrm{ave}}$) of the working fluid in the condensation process will lead to a big error. When the condensation process is equally divided into several small condensation sections, the calculation error of the total performance of the plate condenser can be reduced. In this paper, the condensation process of the working fluid is divided into n small condensation sections, and the average vapor quality ($x_{\mathrm{ave},i}$) of the working fluid in each section is:

(7) $$x_{\mathrm{ave},i}=x_{4}\left(2i-1\right)/2n$$

where $x_{4}$ is the vapor quality of the working fluid at cyclic state point 4, and i equals 1, 2, 3,⋅⋅⋅, n.

Adopting the computation formulas of condensation Nusselt number and friction factor in [19,23,68,69,70], the Nusselt number ($Nu_{\mathrm{wf},\;i}$) and friction factor ($f_{\mathrm{wf},i}$) in each small condensation section of working fluid side are:

(8) $$Nu_{\mathrm{wf},i}=Ge_{1}Re_{\mathrm{eq},\mathrm{wf},i}{}^{Ge_{2}}Pr_{\mathrm{wf},l}{}^{1/3}$$

(9) $$Ge_{1}=11.22{\left(\Lambda /d_{h}\right)}^{-2.83}{\left(\pi \beta /180\right)}^{-4.5}$$

(10) $$Ge_{2}=0.35{\left(\Lambda /d_{h}\right)}^{0.23}{\left(\pi \beta /180\right)}^{1.48}$$

(11) $$f_{\mathrm{wf},i}=Ge_{3}Re_{\mathrm{eq},\mathrm{wf},i}{}^{Ge_{4}}$$

(12) $$Ge_{3}=3521.1{\left(\Lambda /d_{h}\right)}^{4.17}{\left(\pi \beta /180\right)}^{-7.75}$$

(13) $$Ge_{4}=-1.024{\left(\Lambda /d_{h}\right)}^{0.0925}{\left(\pi \beta /180\right)}^{-1.3}$$

where $Pr_{\mathrm{wf},\;l}$ is the Prandtl number of the working fluid in the SLS, and the equivalent Reynolds number $Re_{\mathrm{eq},\mathrm{wf},i}$ of the working fluid in each small condensation section is:

(14) $$Re_{\mathrm{eq},\mathrm{wf},i}=G_{\mathrm{eq},\mathrm{wf},i}d_{h}/\mu_{\mathrm{wf},l}$$

where $\mu_{\mathrm{wf},l}$ is the dynamic viscosity of the working fluid in the SLS, and the equivalent mass flow rate (MFR) $G_{\mathrm{eq},\mathrm{wf},i}$ of the working fluid per cross-sectional area in each small condensation section is:

(15) $$G_{\mathrm{eq},\mathrm{wf},i}=G_{\mathrm{wf}}\left[\left(1-x_{m,i}\right)+x_{m,i}{\left(\rho_{\mathrm{wf},l}/\rho_{\mathrm{wf},v}\right)}^{0.5}\right]$$

where $\rho_{\mathrm{wf},l}$ and $\rho_{\mathrm{wf},v}$ are the densities of the working fluid in the SLS and saturated vapor state, respectively, and MFR $G_{\mathrm{wf}}$ of the working fluid per cross-sectional area is:

(16) $$G_{\mathrm{wf}}={\dot{m}}_{\mathrm{wf}}/\left(N_{\mathrm{wf}}A_{s}\right)$$

where ${\dot{m}}_{\mathrm{wf}}$ is the MFR of the working fluid and $A_{s}$ (equals w⋅b) is the cross-sectional area of the single flow channel in the plate condenser.

According to Reference [70], the surface HTC ($\alpha_{\mathrm{wf},i}$) in each small condensation section of the working fluid side is:

(17) $$\alpha_{\mathrm{wf},\;i}=Nu_{\mathrm{wf},\;i}\lambda_{\mathrm{wf},l}/d_{h}$$

where $\lambda_{\mathrm{wf},l}$ is the thermal conductivity of the working fluid in the SLS. And the average surface HTC ($\alpha_{\mathrm{wf}}$) on the working fluid side is:

(18) $$\alpha_{\mathrm{wf}}=\left(\sum_{i=1}^{n}\alpha_{\mathrm{wf},i}\right)/n$$

The pressure drop ($\Delta p_{\mathrm{wf},i}$) of each small condensation section on the working fluid side can be obtained based on Equations (11)–(16):

(19) $$\Delta p_{\mathrm{wf},i}=2f_{\mathrm{wf},i}\left[L_{\mathrm{eff}}\phi /\left(nd_{h}\right)\right]\left(G_{\mathrm{eq},\mathrm{wf},i}{}^{2}/\rho_{\mathrm{wf},l}\right)$$

### 2.4. Performance of Plate Condenser on Cold Seawater Side

Although the cold seawater absorbs heat, it does not undergo a phase transition and its temperature changes small. Adopting the computation formulas of Nusselt number and friction factor in Reference [71], the Nusselt number ($Nu_{c}$) and friction factor ($f_{c}$) on the cold seawater side are:

(20) $$\begin{array}{ll} Nu_{c}= & \left(0.2668-0.006967\beta +7.244\times 10^{-5}\beta^{2}\right)\times \left(20.78-50.94\phi +41.16\phi^{2}-10.51\phi^{3}\right)\\ & \times Re_{c}{}^{\left[0.728+0.0543\sin\left(\pi \beta /45+3.7\right)\right]}Pr_{c}{}^{1/3}{\left(\mu_{c}/\mu_{w}\right)}^{0.14} \end{array}$$

(21) $$\begin{array}{ll} f_{c}= & \left(2.917-0.1277\beta +2.016\times 10^{-3}\beta^{2}\right)\times \left(5.474-19.02\phi +18.93\phi^{2}-5.341\phi^{3}\right)\\ & \times Re_{c}^{-\left[0.2+0.0577\sin\left(\pi \beta /45+2.1\right)\right]} \end{array}$$

where $Pr_{c}$ and $\mu_{c}$ are the cold-seawater Prandtl number and dynamic viscosity, respectively, and the cold-seawater Reynolds number $Re_{c}$ is:

(22) $$Re_{c}=G_{c}d_{h}/\mu_{c}$$

where the cold-seawater MFR $G_{c}$ per cross-sectional area is:

(23) $$G_{c}={\dot{m}}_{c}/\left(N_{c}A_{s}\right)$$

where ${\dot{m}}_{c}$ is the cold-seawater MFR.

The surface THC ($\alpha_{c}$) and pressure drop ($\Delta p_{c}$) on the cold seawater side are:

(24) $$\alpha_{c}=Nu_{c}\lambda_{c}/d_{h}$$

(25) $$\Delta p_{c}=2f_{c}\left(L_{\mathrm{eff}}\phi /d_{h}\right)\left(G_{c}{}^{2}/\rho_{c}\right)$$

where $\rho_{c}$ is the cold-seawater density.

### 2.5. Overall Performance of Plate Condenser

The total HTC ($K_{\mathrm{cond}}$) of the plate condenser is:

(26) $$K_{\mathrm{cond}}={\left(1/\alpha_{\mathrm{wf}}+R_{\mathrm{wf}}+\delta_{p}/\lambda_{p}+R_{c}+1/\alpha_{c}\right)}^{-1}$$

where $R_{\mathrm{wf}}$ and $R_{c}$ are the fouling resistances on working fluid and cold seawater sides, and $\lambda_{p}$ is the HTP thermal conductivity.

The pumping power ($P_{\mathrm{wf}}$) due to friction loss on the working fluid side is:

(27) $$P_{\mathrm{wf}}={\dot{m}}_{\mathrm{wf}}\sum_{i=1}^{n}\left[\Delta p_{\mathrm{wf},i}/\left(\rho_{\mathrm{wf},i}\eta_{p,\mathrm{wf}}\right)\right]$$

where $\eta_{p,\mathrm{wf}}$ is the efficiency of the working fluid pump, and the density ($\rho_{\mathrm{wf},i}$) of the working fluid in each small condensation section is:

(28) $$\rho_{\mathrm{wf},i}={\left[x_{m,i}/\rho_{\mathrm{wf},v}+\left(1-x_{m,i}\right)/\rho_{\mathrm{wf},l}\right]}^{-1}$$

The pumping power ($P_{c}$) due to friction loss on the cold seawater side is:

(29) $$P_{c}={\dot{m}}_{c}\Delta p_{c}/\left(\rho_{c}\eta_{p,\;c}\right)$$

where $\eta_{p,\;c}$ is the efficiency of the cold seawater pump.

The total pumping power ($P_{\mathrm{sum}}$) due to friction loss can be obtained based on Equations (27)–(29):

(30) $$P_{\mathrm{sum}}=P_{\mathrm{wf}}+P_{c}$$

The plate condenser is treated as an isolated system. For an isolated system with the pure heat transfer process, the entropy generation is:

(31) $$dS_{\mathrm{ios}}=dS_{A}+dS_{B}$$

where $dS_{A}$ and $dS_{B}$ are the entropy changes of the isolated system in heat release and absorption processes, respectively. The entropy change rate (${\dot{S}}_{g,\;\mathrm{wf}}$) of the working fluid in heat release process is:

(32) $${\dot{S}}_{g,\mathrm{wf}}=-{\dot{m}}_{\mathrm{wf}}\left(h_{4}-h_{5}\right)/T_{\mathrm{cond}}$$

And the entropy change rate (${\dot{S}}_{g,c}$) of the cold seawater in heat absorption process is:

(33) $${\dot{S}}_{g,c}={\dot{m}}_{c}C_{p,c}\ln\left(T_{c,\mathrm{out}}/T_{c,\mathrm{in}}\right)$$

Combining Equations (31)–(33) yields the EGR (${\dot{S}}_{g}$) in heat transfer process:

(34) $${\dot{S}}_{g}={\dot{S}}_{g,\mathrm{wf}}+{\dot{S}}_{g,c}={\dot{m}}_{c}C_{p,c}\ln\left(T_{c,\mathrm{out}}/T_{c,\mathrm{in}}\right)-{\dot{m}}_{\mathrm{wf}}\left(h_{4}-h_{5}\right)/T_{\mathrm{cond}}$$

According to the HE theory, the total HTR (${\dot{Q}}_{\mathrm{cond}}$) of the plate condenser is:

(35) $${\dot{Q}}_{\mathrm{cond}}=K_{\mathrm{cond}}A_{\mathrm{sum}}\Delta T_{\mathrm{cond}}={\dot{m}}_{\mathrm{wf}}\left(h_{4}-h_{5}\right)={\dot{m}}_{c}c_{p,c}\left(T_{c,\mathrm{out}}-T_{c,\mathrm{in}}\right)$$

where $c_{p,c}$ is the cold-seawater specific heat, and $\Delta T_{\mathrm{cond}}$ is a logarithmic mean temperature difference. When the plate condenser adopts down-flow configuration, $\Delta T_{\mathrm{cond}}$ can be calculated as:

(36) $$\Delta T_{\mathrm{cond}}=\frac{\left(T_{\mathrm{cond}}-T_{c,\mathrm{in}}\right)-\left(T_{\mathrm{cond}}-T_{c,\mathrm{out}}\right)}{\ln\frac{T_{\mathrm{cond}}-T_{c,\mathrm{in}}}{T_{\mathrm{cond}}-T_{c,\mathrm{out}}}}$$

## 3. Constructal Design for Plate Condenser with Conventional Optimization Method

### 3.1. Optimization Objective of Constructal Design

The plate condenser is an exothermic device in OTEC system. When the heat absorption of OTEC system is constant, the increase of the total HTR (${\dot{Q}}_{\mathrm{cond}}$) of the plate condenser will lead to a decrease in cycle thermal efficiency. Thus, it is inapposite to take ${\dot{Q}}_{\mathrm{cond}}$ as the performance evaluation index of the plate condenser. The total pumping power ($P_{\mathrm{sum}}$) due to friction loss reflects the fluid flow characteristic of the plate condenser, it can be chosen as one of the performance evaluation criteria of the plate condenser. Besides, the EGR (${\dot{S}}_{g}$) [72,73,74,75,76,77,78,79,80] in heat transfer process can also be chosen as one of the performance evaluation criteria because it represents the heat transfer characteristic of the plate condenser. Although the irreversibility due to friction loss will lead to entropy generation, the friction loss has been reflected by $P_{\mathrm{sum}}$. Thus, the EGR due to friction loss is not calculated to avoid double-counting, which is also too small compared with that due to heat transfer.

Singly optimizing $P_{\mathrm{sum}}$ or ${\dot{S}}_{g}$ cannot fully reflect the performance of the plate condenser. Therefore, a composite function (CF) which synthetically considers $P_{\mathrm{sum}}$ and ${\dot{S}}_{g}$ is established by applying the linear weighting method [81,82,83]:

(37) $$F_{\mathrm{SP}}=a_{0}\frac{{\dot{S}}_{g}}{{\dot{S}}_{g,\mathrm{int}}}+\left(1-a_{0}\right)\frac{P_{\mathrm{sum}}}{P_{\mathrm{sum},\mathrm{int}}}$$

where $a_{0}$ is the weighting coefficient, and $P_{\mathrm{sum},\mathrm{int}}$ and ${\dot{S}}_{g,\mathrm{int}}$ are the pumping power and EGR calculated by the initial design of the plate condenser. $F_{\mathrm{SP}}$ reflects the comprehensive performance of the plate condenser. When the effective volume ($V_{\mathrm{eff}}$) and total HTR (${\dot{Q}}_{\mathrm{cond}}$) of the plate condenser are fixed, $F_{\mathrm{SP}}$ is a function of the HTP effective length ($L_{\mathrm{eff}}$), width (w), and effective number ($N_{\mathrm{eff}}$). The constructal design of the plate condenser will be conducted by employing $F_{\mathrm{SP}}$ as an optimization objective and $L_{\mathrm{eff}}$, w, and $N_{\mathrm{eff}}$ as optimal design variables. Furthermore, the influences of the structural parameters and $a_{0}$ on CORs will be analyzed.

### 3.2. Optimization Procedure of Constructal Design

The constraint relationship between the condensation temperature ($T_{\mathrm{cond}}$) and total HTC ($K_{\mathrm{cond}}$) can be established based on Equation (35) when the total HTR (${\dot{Q}}_{\mathrm{cond}}$) of the plate condenser and MFR (${\dot{m}}_{c}$) of the cold seawater are given. Then $K_{\mathrm{cond}}$ and $T_{\mathrm{cond}}$ can be obtained according to the constraint of the fixed effective volume ($V_{\mathrm{eff}}$) of the plate condenser. And then the MFR (${\dot{m}}_{\mathrm{wf}}$) of the working fluid, total pumping power ($P_{\mathrm{sum}}$) due to friction loss, and EGR (${\dot{S}}_{g}$) in heat transfer process can be obtained. Table 1 lists the values of some parameters in the calculations. To perform the constructal design for the plate condenser, the optimization procedure with conventional single-objective optimization method is given as follows:

(1) The single variable optimization is carried out. The relationship between the CF ($F_{\mathrm{SP}}$) and HTP effective length ($L_{\mathrm{eff}}$) is obtained with the given HTP width (w) and effective number ($N_{\mathrm{eff}}$). The relationships between $F_{\mathrm{SP}}$ and w as well as between $F_{\mathrm{SP}}$ and $N_{\mathrm{eff}}$ are obtained by applying a similar method.
(2) The double variable optimization is carried out by releasing the constraint of w on the basis of singly optimizing $L_{\mathrm{eff}}$. The relationships among $F_{\mathrm{SP}}$, $L_{\mathrm{eff}}$, and w are obtained with the given $N_{\mathrm{eff}}$.
(3) The three variable optimization is carried out by releasing constraint of $N_{\mathrm{eff}}$ on the basis of step 2. The relationships among $F_{\mathrm{SP}}$, $L_{\mathrm{eff}}$, w, and $N_{\mathrm{eff}}$ are obtained.
(4) On the basis of step 3, the CORs of the plate condenser with different structural parameters and weighting coefficient are obtained. The subscripts “m” and “mm” mean the primary and twice minimizations, respectively, and the subscripts “opt” and “oo” mean the primary and twice optimizations, respectively.

The optimizations mentioned above are performed in MATLAB software, in which the ‘fsolve’ function is used.

### 3.3. Results of Constructal Designs

#### 3.3.1. Single Variable Optimization

**Figure 5:**
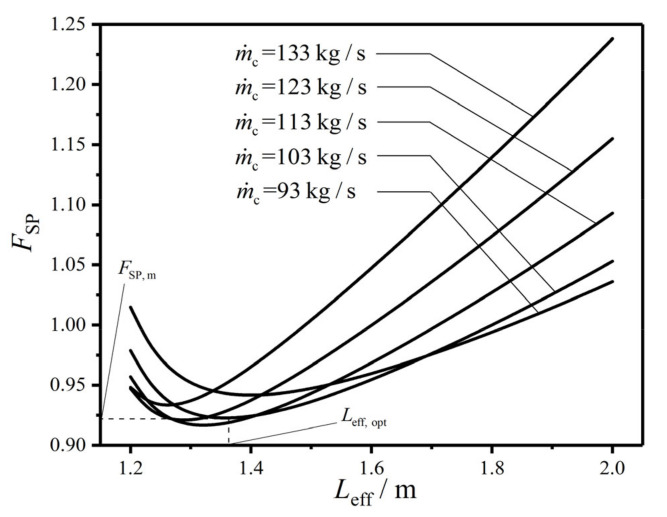
Effect of ${\dot{m}}_{c}$ on $F_{\mathrm{SP}}$ versus $L_{\mathrm{eff}}$.

**Figure 6:**
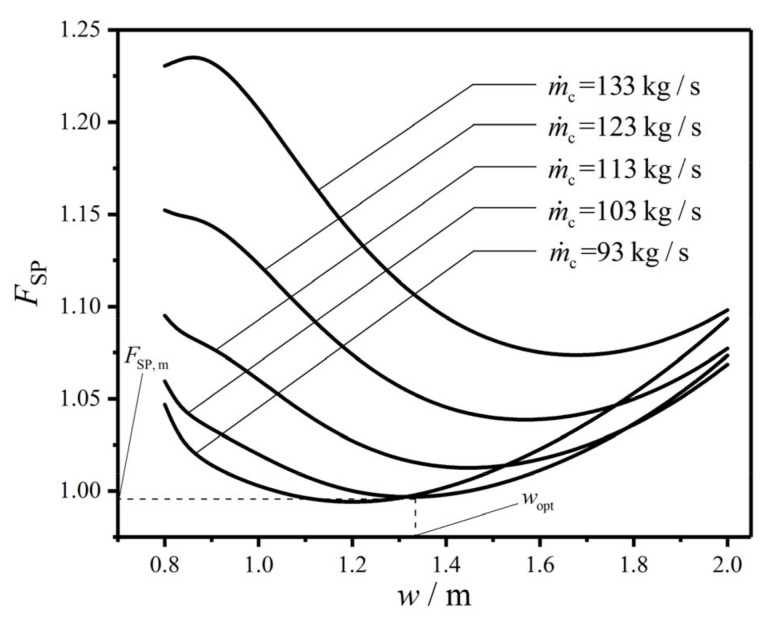
Effect of ${\dot{m}}_{c}$ on $F_{\mathrm{SP}}$ versus w.

**Figure 7:**
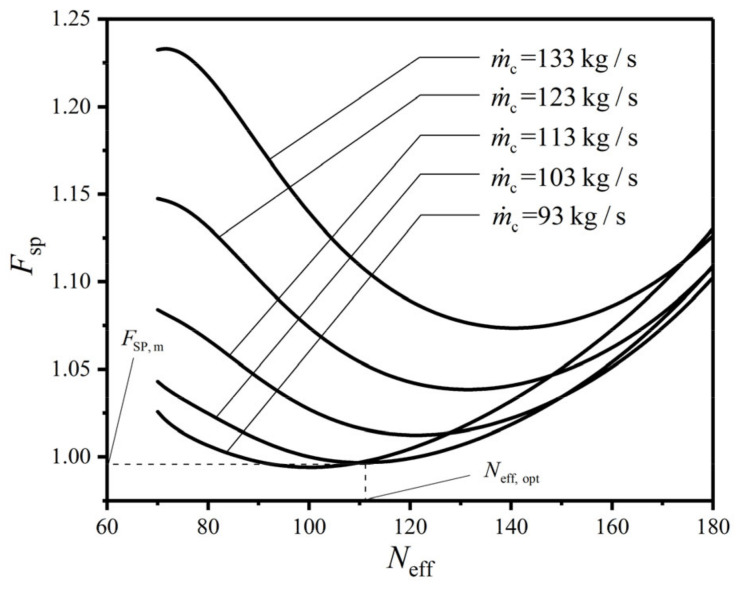
Effect of ${\dot{m}}_{c}$ on $F_{\mathrm{SP}}$ versus $N_{\mathrm{eff}}$.

Figure 5, Figure 6 and Figure 7 reflect the effects of MFR (${\dot{m}}_{c}$) of the cold seawater on the characteristics of CF ($F_{\mathrm{SP}}$) versus HTP effective length ($L_{\mathrm{eff}}$), $F_{\mathrm{SP}}$ versus HTP width (w), and $F_{\mathrm{SP}}$ versus HTP effective number ($N_{\mathrm{eff}}$), respectively. In the figures, no matter what value ${\dot{m}}_{c}$ takes, $F_{\mathrm{SP}}$ always has the minimum value. Namely, with a given ${\dot{m}}_{c}$, there is always a primary optimal HTP effective length ($L_{\mathrm{eff},\mathrm{opt}}$), a primary optimal HTP width ($w_{\mathrm{opt}}$), and a primary optimal HTP effective number ($N_{\mathrm{eff},\mathrm{opt}}$) to make $F_{\mathrm{SP}}$ reach the primary minimum value ($F_{\mathrm{SP},m}$), respectively. And with the increase of ${\dot{m}}_{c}$, $L_{\mathrm{eff},\mathrm{opt}}$ gradually decreases, while the corresponding $F_{\mathrm{SP},m}$ first diminishes and then augments. It infers that there is an optimal MFR (${\dot{m}}_{c,\mathrm{opt}}$) of the cold seawater to make $F_{\mathrm{SP},m}$ reach the twice minimum value ($F_{\mathrm{SP},\mathrm{mm}}$). At the same time, with the increase of ${\dot{m}}_{c}$, $w_{\mathrm{opt}}$, and $N_{\mathrm{eff},\mathrm{opt}}$ gradually increase, and their corresponding $F_{\mathrm{SP},m}$ also gradually increase. These indicate that the influences of ${\dot{m}}_{c}$ on $w_{\mathrm{opt}}$, $N_{\mathrm{eff},\mathrm{opt}}$, and their corresponding $F_{\mathrm{SP},m}$ are different from those of ${\dot{m}}_{c}$ on $L_{\mathrm{eff},\mathrm{opt}}$ and its corresponding $F_{\mathrm{SP},m}$. Furthermore, with the increase of ${\dot{m}}_{c}$, the curve of $F_{\mathrm{SP}}-L_{\mathrm{eff}}$ maintains a similar trend, while the curves of $F_{\mathrm{SP}}-w$ and $F_{\mathrm{SP}}-N_{\mathrm{eff}}$ gradually appear the maximum values, which significantly increase with the increase of ${\dot{m}}_{c}$. These results show that ${\dot{m}}_{c}$ has both qualitative and quantitative influences on the curves of $F_{\mathrm{SP}}-w$ and $F_{\mathrm{SP}}-N_{\mathrm{eff}}$, and only has a quantitative effect on the curve of $F_{\mathrm{SP}}-L_{\mathrm{eff}}$. The explanation is that as ${\dot{m}}_{c}$ increases, the total pumping power ($P_{\mathrm{sum}}$) due to friction loss augments while the EGR (${\dot{S}}_{g}$) in heat transfer process diminishes, which causes the shape of $F_{\mathrm{SP}}$ curve to gradually approximate $P_{\mathrm{sum}}$ curve and keep away from ${\dot{S}}_{g}$ curve. Besides the above reason, it can be found from the calculations that the curves of $P_{\mathrm{sum}}-L_{\mathrm{eff}}$ and ${\dot{S}}_{g}-L_{\mathrm{eff}}$ are both monotones. Both the curves of ${\dot{S}}_{g}-w$ and ${\dot{S}}_{g}-N_{\mathrm{eff}}$ only have the minimum points, and the curves of $P_{\mathrm{sum}}-w$ and $P_{\mathrm{sum}}-N_{\mathrm{eff}}$ both have the maximum and minimum points. Therefore, ${\dot{m}}_{c}$ only has a quantitative influence on $F_{\mathrm{SP}}-L_{\mathrm{eff}}$ curve, while it has both quantitative and qualitative influences on the curves of $F_{\mathrm{SP}}-w$ and $F_{\mathrm{SP}}-N_{\mathrm{eff}}$.

When ${\dot{m}}_{c}$ is equal to 103 kg/s, $F_{\mathrm{SP}}$ first decreases and then increases with the increases of $L_{\mathrm{eff}}$, w, and $N_{\mathrm{eff}}$ in Figure 5, Figure 6 and Figure 7. There is a primary optimal HTP effective length ($L_{\mathrm{eff},\mathrm{opt}}=1.350\;m$), a primary optimal HTP width ($w_{\mathrm{opt}}=1.325\;m$), and a primary optimal HTP effective number ($N_{\mathrm{eff},\mathrm{opt}}=110$) to make $F_{\mathrm{SP}}$ reach $F_{\mathrm{SP},m}$, which are 0.922, 0.997, and 0.997, respectively. And they diminish by 7.8%, 0.3%, and 0.3% compared with the initial design of plate condenser. Thus, the result of singly optimizing $L_{\mathrm{eff}}$ is superior to those of singly optimizing w and $N_{\mathrm{eff}}$. The explanation for the decrease of $F_{\mathrm{SP}}$ is that $P_{\mathrm{sum}}$ diminishes 46.64%, 9.25%, and 7.47%, respectively, while ${\dot{S}}_{g}$ augments 5.21%, 2.66%, and 2.04%, respectively. The decrease of $P_{\mathrm{sum}}$ is the main contribution to the decrease of $F_{\mathrm{SP}}$. Finally, the plate condenser after constructal optimization greatly reduces the total flow pressure drops, and improve its comprehensive performance.

#### 3.3.2. Double Variable Optimization

**Figure 8:**
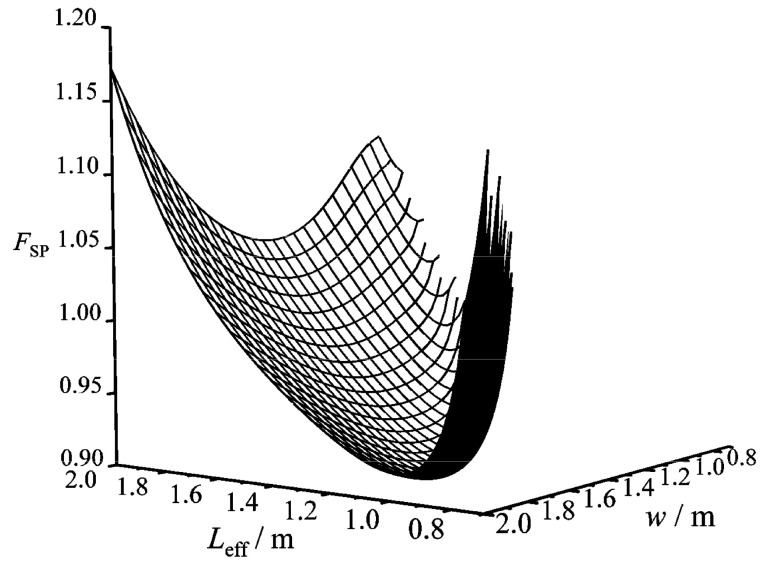
Three-dimensional diagram of $F_{\mathrm{SP}}$ versus $L_{\mathrm{eff}}$ and w.

**Figure 9:**
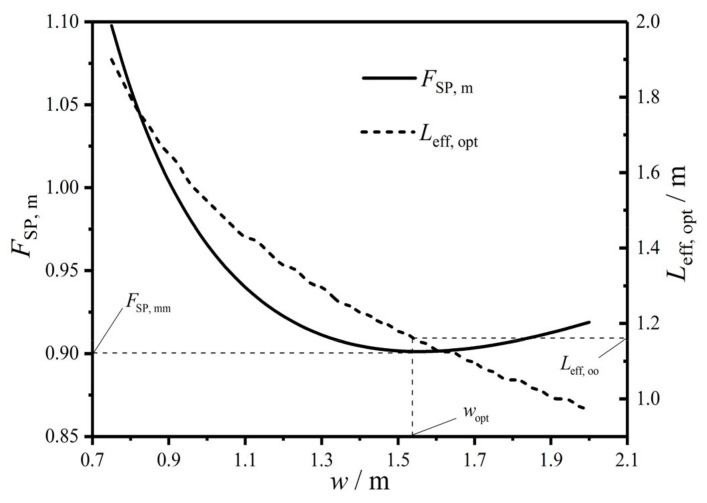
Relationships of $F_{\mathrm{SP},\;m}$ and $L_{\mathrm{eff},\;\mathrm{opt}}$ versus w.

Figure 8 gives a three-dimensional diagram of the CF ($F_{\mathrm{SP}}$) versus effective length ($L_{\mathrm{eff}}$) and width (w) of the HTP. In the figure, there is always a primary optimal HTP effective length ($L_{\mathrm{eff},\mathrm{opt}}$) to make $F_{\mathrm{SP}}$ reach the primary minimum ($F_{\mathrm{SP},m}$) for any $F_{\mathrm{SP}}-L_{\mathrm{eff}}$ curve with a given w. Figure 9 gives two-dimensional relationships between $F_{\mathrm{SP},m}$ and w as well as between $L_{\mathrm{eff},\mathrm{opt}}$ and w. One can know from the figure that as w increases, $F_{\mathrm{SP},m}$ first diminishes and then augments, while $L_{\mathrm{eff},\mathrm{opt}}$ monotonically decreases. There is a primary optimal HTP width ($w_{\mathrm{opt}}=1.55\;m$) to make $F_{\mathrm{SP},m}$ be the twice minimum ($F_{\mathrm{SP},\mathrm{mm}}=0.901$), and the twice optimal HTP effective length ($L_{\mathrm{eff},\mathrm{oo}}$) is 1.15 m. Compared with the plate condenser after singly optimizing $L_{\mathrm{eff}}$, $F_{\mathrm{SP}}$ after simultaneously optimizing $L_{\mathrm{eff}}$ and w diminishes 2.3%. The reason for the decrease of $F_{\mathrm{SP}}$ is that the total pumping power ($P_{\mathrm{sum}}$) due to friction loss diminishes 19.82%, while the EGR (${\dot{S}}_{g}$) in heat transfer process augments 3.77%. Thus, the decrease of $P_{\mathrm{sum}}$ is the main contribution to the decrease of $F_{\mathrm{SP}}$. Besides, the result also indicates that further optimizing w on the basis of singly optimizing $L_{\mathrm{eff}}$ can significantly improve the comprehensive performance of the plate condenser.

#### 3.3.3. Three Variable Optimization

**Figure 10:**
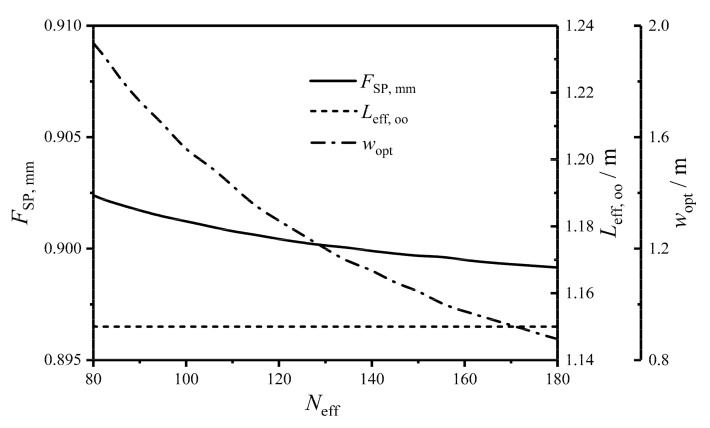
Relationships of $F_{\mathrm{SP},\;\mathrm{mm}}$, $L_{\mathrm{eff},\;\mathrm{oo}}$, and $w_{\mathrm{opt}}$ versus $N_{\mathrm{eff}}$.

**Figure 11:**
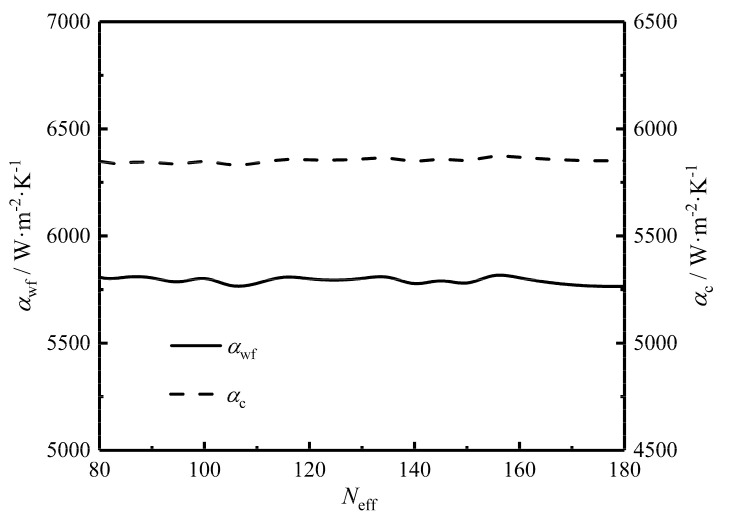
Relationships of $\alpha_{wf}$ and $\alpha_{c}$ versus $N_{\mathrm{eff}}$.

Figure 10 gives the relationships between the twice minimum CF ($F_{\mathrm{SP},\mathrm{mm}}$) and HTP effective number ($N_{\mathrm{eff}}$), between the twice optimal HTP effective length ($L_{\mathrm{eff},\mathrm{oo}}$) and $N_{\mathrm{eff}}$, and between the primary optimal HTP width ($w_{\mathrm{opt}}$) and $N_{\mathrm{eff}}$. One can know from the figure that with the increase of $N_{\mathrm{eff}}$, $L_{\mathrm{eff},\mathrm{oo}}$ remains stable, while $w_{\mathrm{opt}}$ monotonically decreases and has a wide variation. $F_{\mathrm{SP},\mathrm{mm}}$ slowly diminishes with the growth of $N_{\mathrm{eff}}$, and the variation of $F_{\mathrm{SP},\mathrm{mm}}$ is small. The results show that further optimizing $N_{\mathrm{eff}}$ on the basis of the twice optimization cannot significantly improve the comprehensive performance of the plate condenser. When $N_{\mathrm{eff}}=135$, $F_{\mathrm{SP},\mathrm{mm}}$ is equal to 0.900, and when $N_{\mathrm{eff}}=180$, $F_{\mathrm{SP},\mathrm{mm}}$ is equal to 0.899. $F_{\mathrm{SP},\mathrm{mm}}$ has little change in the range of $135<N_{\mathrm{eff}}<180$. Thus, it is meaningless to further increase the HTP effective number when $N_{\mathrm{eff}}\;>\;135$. The reasons for the low dependency of $N_{\mathrm{eff}}$ on $F_{\mathrm{SP},\mathrm{mm}}$ are as follows: (1) $N_{\mathrm{eff}}$ has little effects on HTCs of the HTP on the working fluid and cold seawater sides, as shown in Figure 11. Thus, the EGR (${\dot{S}}_{g}$) which reflects the heat transfer characteristic of the plate condenser has a small effect. (2) $N_{\mathrm{eff}}$ has little effects on the MFRs ($G_{\mathrm{wf}}$ and $G_{c}$) of the working fluid and cold-seawater per cross-sectional area because $L_{\mathrm{eff},\mathrm{oo}}$ remains stable and the effective volume ($V_{\mathrm{eff}}$) is fixed. $G_{\mathrm{wf}}$, $G_{c}$, and $L_{\mathrm{eff}}$ are three key parameters to determine the fluid flow characteristic of the plate condenser. Their small changes make the total pumping power ($P_{\mathrm{sum}}$) less affected by $N_{\mathrm{eff}}$.

#### 3.3.4. Effects of Design Parameters on Optimization Results

**Figure 12:**
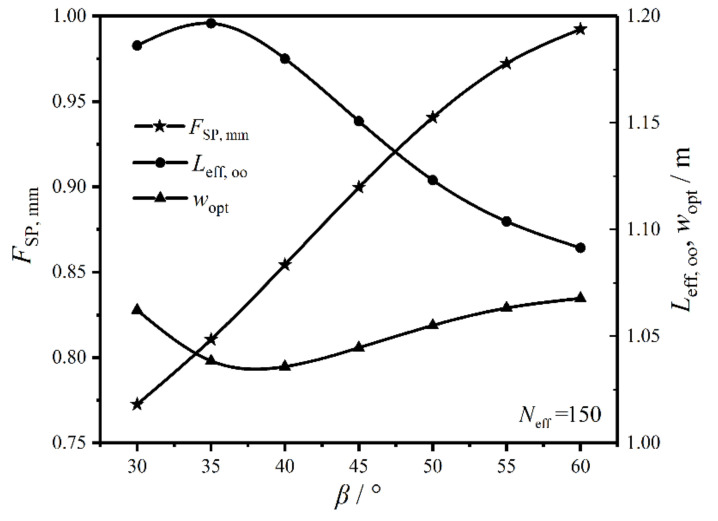
Relationships of $F_{\mathrm{SP},\mathrm{mm}}$, $L_{\mathrm{eff},\mathrm{oo}}$, and $w_{\mathrm{opt}}$ versus β.

Figure 12 gives the relationships of the twice minimum CF ($F_{\mathrm{SP},\mathrm{mm}}$), twice optimal HTP effective length ($L_{\mathrm{eff},\mathrm{oo}}$), and primary optimal HTP width ($w_{\mathrm{opt}}$) versus HTP corrugation angle (β) when $N_{\mathrm{eff}}$ equals to 150. One can know from the figure that $F_{\mathrm{SP},\mathrm{mm}}$ grows with the increase of β, and the optimal performance is significantly affected by β. As β increases, the fluid distribution tends to be uniform, and the fluid flow along the corrugating trough decreases. Moreover, with the increase of β, the three-dimensional turbulence forms, and the eddy current density gradually increases. These reasons make the pressure drops of the working fluid and cold seawater increase, which leads to the augmentation of $F_{\mathrm{SP},\mathrm{mm}}$. On the other hand, the fluid flow state transits from the crossflow to zigzag flow with the increase of β, leading to the enhancement of turbulence intensity and the increase of the total HTC. Thus, the outlet temperature ($T_{c,\mathrm{out}}$) of the cold seawater increases when the total HTR (${\dot{Q}}_{\mathrm{cond}}$) of the plate condenser is fixed. With the increase of $T_{c,\mathrm{out}}$, the EGR (${\dot{S}}_{g}$) in heat transfer process increases and $F_{\mathrm{SP},\mathrm{mm}}$ further augments. With the increase of β, the variation trend of $L_{\mathrm{eff},\mathrm{oo}}$ is opposite to that of $w_{\mathrm{opt}}$. When β varies from $30^{o}$ to $35^{o}$, $L_{\mathrm{eff},\mathrm{oo}}$ gradually increases, while $w_{\mathrm{opt}}$ gradually decreases. When β varies from $40^{o}$ to $60^{o}$, $L_{\mathrm{eff},\mathrm{oo}}$ gradually decreases, while $w_{\mathrm{opt}}$ gradually increases, and the variation of $L_{\mathrm{eff},\mathrm{oo}}$ is larger than that of $w_{\mathrm{opt}}$. Therefore, β has significant impacts on the CORs of the plate condenser.

**Figure 13:**
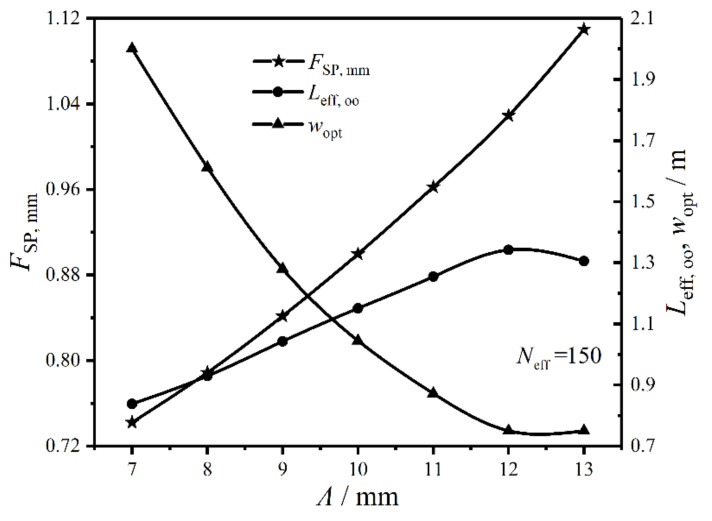
Relationships of $F_{\mathrm{SP},\;\mathrm{mm}}$, $L_{\mathrm{eff},\;\mathrm{oo}}$, and $w_{\mathrm{opt}}$ versus Λ.

Figure 13 gives the relationships of the twice minimum CF ($F_{\mathrm{SP},\mathrm{mm}}$), twice optimal HTP effective length ($L_{\mathrm{eff},\mathrm{oo}}$), and primary optimal HTP width ($w_{\mathrm{opt}}$) versus HTP corrugation wavelength (Λ) under the condition of $N_{\mathrm{eff}}=150$. In the figure, $F_{\mathrm{SP},\mathrm{mm}}$ near linearly augments with the enlargement of Λ, which demonstrates Λ can significantly affect the optimal performance of the plate condenser. The reason is that Λ can make condensation temperature ($T_{\mathrm{cond}}$) increase, and the entropy change rate of the working fluid in heat transfer process increases, which causes $F_{\mathrm{SP},\mathrm{mm}}$ to augment. When Λ varies from 7 mm to 12 mm, $L_{\mathrm{eff},\mathrm{oo}}$ gradually increases, while $w_{\mathrm{opt}}$ significantly decreases. When Λ varies from 12 mm to 13 mm, $L_{\mathrm{eff},\mathrm{oo}}$ sharply decreases, while $w_{\mathrm{opt}}$ remains stable. Therefore, Λ has great influences on the CORs of the plate condenser.

**Figure 14:**
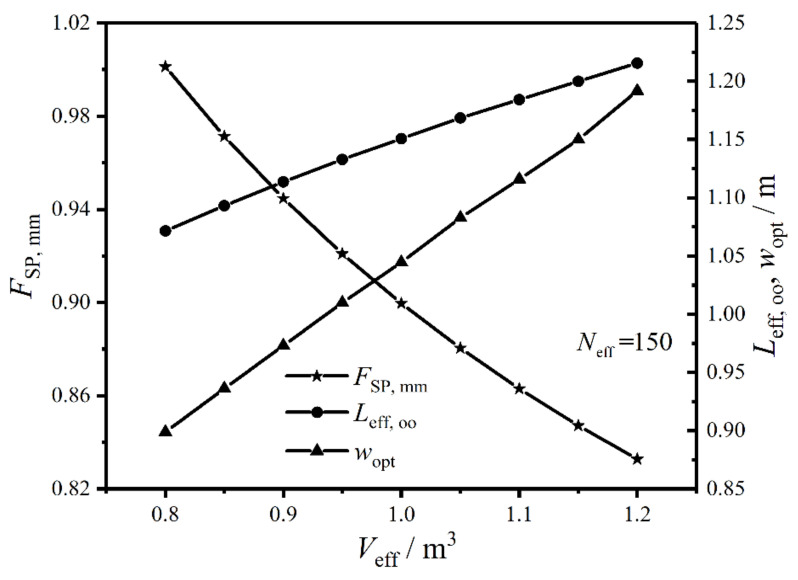
Relationships of $F_{\mathrm{SP},\;\mathrm{mm}}$, $L_{\mathrm{eff},\;\mathrm{oo}}$, and $w_{\mathrm{opt}}$ versus $V_{\mathrm{eff}}$.

Figure 14 gives the relationships of the twice minimum CF ($F_{\mathrm{SP},\mathrm{mm}}$), twice optimal HTP effective length ($L_{\mathrm{eff},\mathrm{oo}}$), and primary optimal HTP width ($w_{\mathrm{opt}}$) versus effective volume ($V_{\mathrm{eff}}$) of the plate condenser under the condition of $N_{\mathrm{eff}}=150$. In the figure, $F_{\mathrm{SP},\mathrm{mm}}$ near linearly diminishes with the increase of $V_{\mathrm{eff}}$, which demonstrates the comprehensive performance of the plate condenser can be enhanced by increasing $V_{\mathrm{eff}}$. The reason is that the cross-sectional area enlarges with the increase of $V_{\mathrm{eff}}$, which leads to reducing the flow pressure drops and EGR (${\dot{S}}_{g}$) in heat transfer process. When $V_{\mathrm{eff}}$ increases from $0.8\;m^{3}$ to $1.2\;m^{3}$, $L_{\mathrm{eff},\mathrm{oo}}$ and $w_{\mathrm{opt}}$ gradually increase, and the variation trend of the latter is larger than that of the former. Therefore, $V_{\mathrm{eff}}$ has great effects on $L_{\mathrm{eff},\mathrm{oo}}$ and $w_{\mathrm{opt}}$. Moreover, it has a remarkable influence on the optimal performance of the plate condenser, and the main reason is that ${\dot{S}}_{g}$ and the total pumping power ($P_{\mathrm{sum}}$) due to friction loss vary in the same direction with the increase of $w_{\mathrm{opt}}$.

**Figure 15:**
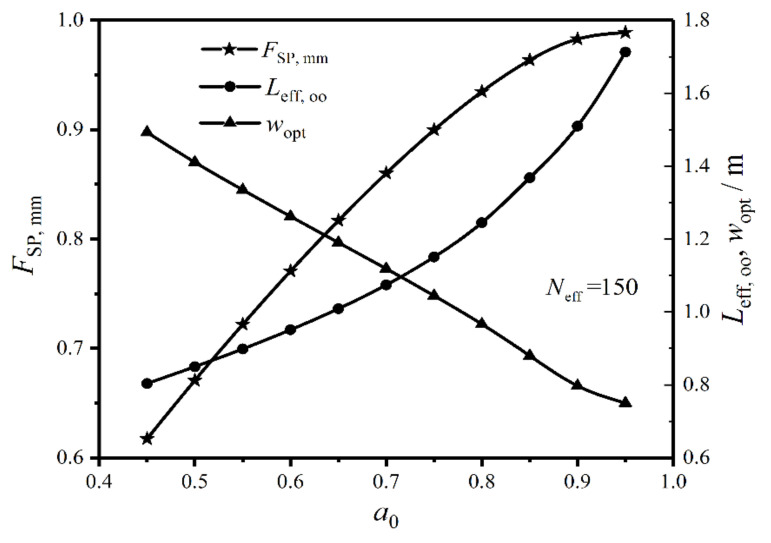
Relationships of $F_{\mathrm{SP},\;\mathrm{mm}}$, $L_{\mathrm{eff},\;\mathrm{oo}}$, and $w_{\mathrm{opt}}$ versus $a_{0}$.

Figure 15 gives the relationships of the twice minimum CF ($F_{\mathrm{SP},\mathrm{mm}}$), twice optimal HTP effective length ($L_{\mathrm{eff},\mathrm{oo}}$), and primary optimal HTP width ($w_{\mathrm{opt}}$) versus weighting coefficient ($a_{0}$) under the condition of $N_{\mathrm{eff}}=150$. One can obtain from the figure that the plate condenser has the optimal performance and optimal construct in the range of $0.45\leq a_{0}\leq 0.95$. When $a_{0}$ varies from 0.45 to 0.95, the proportion of EGR (${\dot{S}}_{g}$) in heat transfer process increases, and the proportion of total pumping power ($P_{\mathrm{sum}}$) due to friction loss decreases. With the increase of $a_{0}$, $F_{\mathrm{SP},\mathrm{mm}}$ augments and its variation amplitude gradually diminishes, $L_{\mathrm{eff},\mathrm{oo}}$ increases and its variation amplitude gradually increases, while $w_{\mathrm{opt}}$ near linearly decreases. Therefore, $a_{0}$ has remarkable influences on the CORs of the plate condenser.

All of the above research is important and helpful for OTEC system. The obtained optimal performance of the plate condenser can provide guidelines for the overall performance evaluation of OTEC system, and the obtained optimal construct of the plate condenser can be applied to design the plate condensers. Moreover, the above research considers the actual situation in which some parameters may deviate from the specific values, and analyze the impacts of variable parameters on the CORs. The obtained optimization results with different parameters can provide wide guidelines to design condensers under different operating conditions.

## 4. Constructal Design for Plate Condenser with Multi-Objective Genetic Algorithm

The above studies take the linear weighting method, which converts two optimization objectives into a single optimization objective, to solve the multi-objective optimization issue. However, the optimization objective may not be a linear weighting of two objective functions. To provide a wider selection for optimization and make the calculation results guide the designs of the plate condensers better, a multi-objective genetic algorithm [84,85,86,87] that is an intelligent algorithm provided by MATLAB software is adopted further.

### 4.1. Optimization Procedure of Multi-Objective Genetic Algorithm

**Figure 16:**
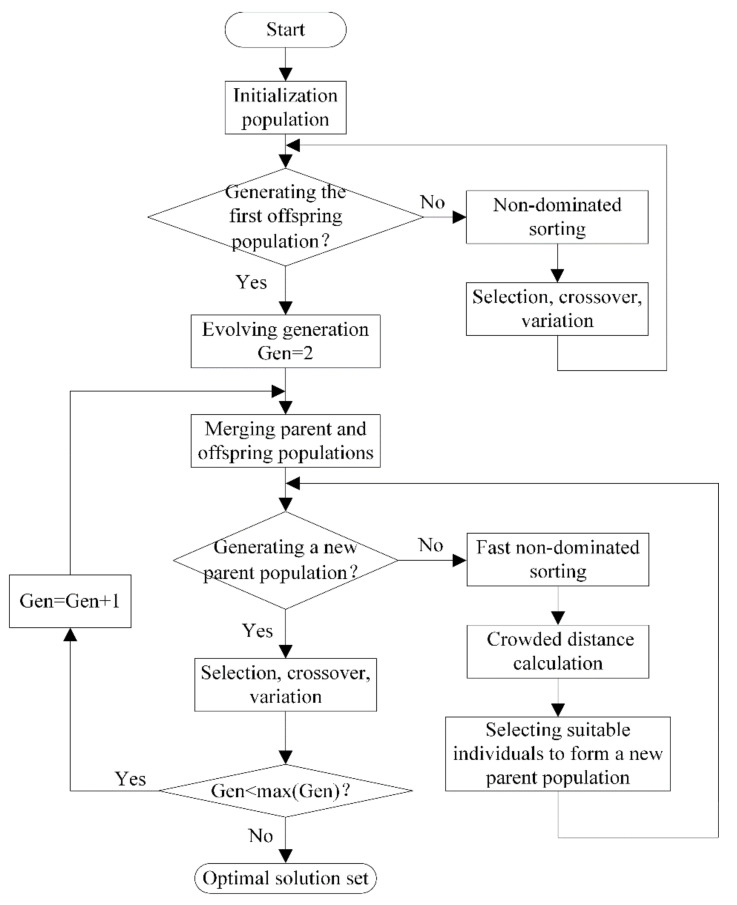
Flowchart of non-dominated sorting genetic algorithm II (NSGA-II).

The non-dominated sorting genetic algorithm II (NSGA-II), which is one of the multi-objective optimization algorithms, has strong points of high operation efficiency and good distribution of solution sets. Besides, NSGA-II is good at solving the low-dimensional optimization problem. In this paper, the optimal design variables are $L_{\mathrm{eff}}$ and w, thus, NSGA-II is very suitable. Figure 16 gives a flowchart of NSGA-II, in which the following parameters are set: the size of the population is set as 300, the evolution generation is set as 500, and “PlotFcns” is chosen as “gaplotpareto”. The basic idea of NSGA-II is given as follows:

(1) An initial population with N scale is randomly generated, and the first offspring population is obtained through selection, crossover, and variation after non-dominated sorting.
(2) The parent and offspring populations start to merge from the second generation. At the same time of performing the fast non-dominated sorting, the crowded distance of each individual in the non-dominated layer is calculated. A new parent population is formed by selecting suitable individuals based on the non-dominated relationship and the crowded distance of the individual.
(3) A new offspring population is generated through selection, crossover, and variation of the parent population, and to circulate repeatedly until the ending conditions are satisfied. In this paper, the size of the population is set as 300, the evolution generation is set as 500, and “PlotFcns” is chosen as “gaplotpareto”.

### 4.2. Results of Constructal Design

**Figure 17:**
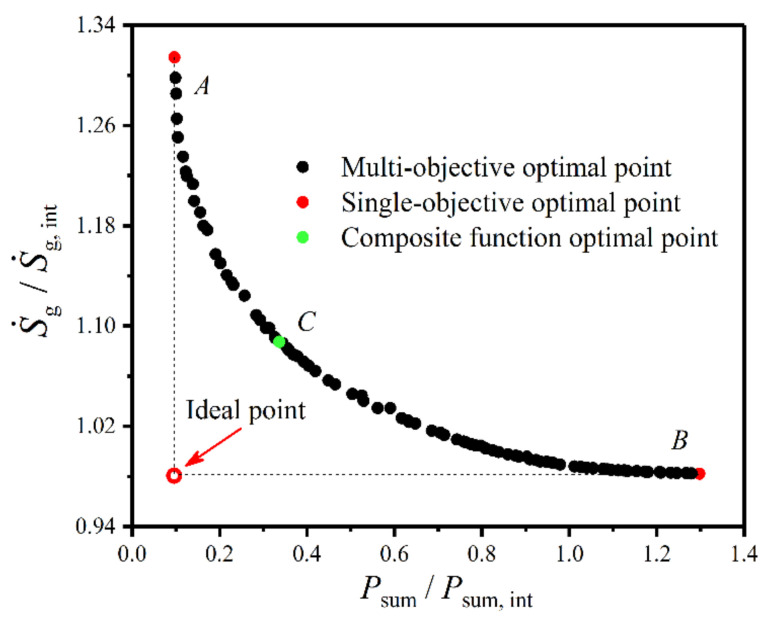
Pareto front of the plate condenser based on multi-objective optimization.

Figure 17 gives Pareto front of the plate condenser based on multi-objective optimization of the minimum dimensionless EGR (${\dot{S}}_{g}/{\dot{S}}_{g,\mathrm{int}}$) and minimum dimensionless total pumping power ($P_{\mathrm{sum}}/P_{\mathrm{sum},\mathrm{int}}$). One can know from the figure that the minimum $P_{\mathrm{sum}}/P_{\mathrm{sum},\mathrm{int}}$ and the minimum ${\dot{S}}_{g}/{\dot{S}}_{g,\mathrm{int}}$ are two conflicting and influencing objective functions for the plate condenser. Each point in Pareto front is an optimal solution for the plate condenser, and at least one objective function ($P_{\mathrm{sum}}/P_{\mathrm{sum},\mathrm{int}}$ or ${\dot{S}}_{g}/{\dot{S}}_{g,\mathrm{int}}$) of the Pareto optimal solution is better than that of the solution outside the Pareto set. Namely, if $P_{\mathrm{sum}}/P_{\mathrm{sum},\mathrm{int}}$ (or ${\dot{S}}_{g}/{\dot{S}}_{g,\mathrm{int}}$) decreases on the basis of the optimal solution, ${\dot{S}}_{g}/{\dot{S}}_{g,\mathrm{int}}$ (or $P_{\mathrm{sum}}/P_{\mathrm{sum},\mathrm{int}}$) must be increased. Therefore, the optimal solution in the Pareto front has the least objective conflict compared with other solutions. In the figure, the ideal point at the lower-left corner is the minimum point that $P_{\mathrm{sum}}/P_{\mathrm{sum},\mathrm{int}}$ or ${\dot{S}}_{g}/{\dot{S}}_{g,\mathrm{int}}$ should reach, but it cannot be reached in engineering because $P_{\mathrm{sum}}/P_{\mathrm{sum},\mathrm{int}}$ and ${\dot{S}}_{g}/{\dot{S}}_{g,\mathrm{int}}$ cannot reach minimums simultaneously. Points A and B can be regarded as the optimal solutions of independently optimizing $P_{\mathrm{sum}}/P_{\mathrm{sum},\mathrm{int}}$ and ${\dot{S}}_{g}/{\dot{S}}_{g,\mathrm{int}}$, respectively. Point C is the optimal solution of the plate condenser with CF ($F_{\mathrm{SP}}$) as an optimization objective, and it is also one of the optimal solutions in the Pareto front. Therefore, the Pareto front provides better selections for performance optimizations of the plate condenser.

## 5. Conclusions

The condenser is a crucial component in an OTEC system. This paper applies constructal theory to conduct constructal optimization for the plate condenser by optimizing the HTP effective length ($L_{\mathrm{eff}}$), width (w), and effective number ($N_{\mathrm{eff}}$). The CORs of the plate condenser are obtained. The effects of different parameters on the CORs are analyzed and compared. Moreover, Pareto optimal set of the plate condenser is depicted by using NSGA-II. The obtained results are:

(1) There is a primary optimal HTP effective length ($L_{\mathrm{eff},\mathrm{opt}}=1.350\;m$), a primary optimal HTP width ($w_{\mathrm{opt}}=1.325\;m$), and a primary optimal HTP effective number ($N_{\mathrm{eff},\mathrm{opt}}=110$) to make $F_{\mathrm{SP}}$ respectively reach 0.922, 0.997, and 0.997. $L_{\mathrm{eff}}$ has a more significant effect than w and $N_{\mathrm{eff}}$, and it can be chosen as the main design parameter to improve the performance of the plate condenser.
(2) Continuing to optimize w on the basis of singly optimizing $L_{\mathrm{eff}}$ can partly improve the comprehensive performance of the plate condenser. The twice minimum CF ($F_{\mathrm{SP},\mathrm{mm}}$) after simultaneously optimizing $L_{\mathrm{eff}}$ and w is 0.901, which is 2.3% less than $F_{\mathrm{SP},m}$ after singly optimizing $L_{\mathrm{eff}}$. The twice optimal HTP effective length ($L_{\mathrm{eff},\mathrm{oo}}$) and $w_{\mathrm{opt}}$ are 1.15 m and 1.55 m, respectively.
(3) Further optimizing the HTP effective number ($N_{\mathrm{eff}}$) on the basis of twice optimization cannot significantly improve the comprehensive performance of the plate condenser. The corrugation angle (β), corrugation wavelength (Λ), effective volume ($V_{\mathrm{eff}}$), and weighting coefficient ($a_{0}$) have different influences on the optimal performance and optimal construct. $F_{\mathrm{SP},\mathrm{mm}}$ gradually augments with the increases of β, Λ, and $a_{0}$, and gradually diminishes with the increase of $V_{\mathrm{eff}}$.
(4) Pareto optimal set can provide better choices for the performance optimizations of the plate condenser.
(5) $L_{\mathrm{eff}}$ and w are two important parameters of the plate condenser. Single, double, and three variable optimizations, as well as the Pareto optimal set, all provide the optimal design values of the plate condenser, and they can be an important basis and criteria for designers to design the plate condensers.

The thermal efficiency and power output are two important performance indexes for OTEC system, and they are interesting to researchers. However, this paper took the plate condenser as a research objective, thus only the performance of the plate condenser was optimized. The influences of the plate condenser on the overall performance of OTEC system will be studied in the future system study. Moreover, the research methods and obtained conclusions herein are important for overall performance evaluation and experimental platform establishment of OTEC system. To test and verify these optimization results, the related experiment will be carried out in the next studies.

## Figures and Tables

**Table 1:** Values and variation ranges of partial parameters.

Parameters	Notations	Values	Variation Ranges	Units
Initial pumping power due to friction loss	$P_{\mathrm{sum},\;\mathrm{int}}$	6728.42	-	W
Initial EGR in heat transfer process	${\dot{S}}_{g,\;\mathrm{int}}$	30.85	-	W/K
Total HTR of the plate condenser	${\dot{Q}}_{\mathrm{cond}}$	1.25 × 10^6^	-	W
Evaporation pressure of working fluid in the evaporator	$p_{\mathrm{eva}}$	991.85	-	kPa
Temperature of cold seawater at the inlet of the plate condenser	$T_{c,\;\mathrm{in}}$	277.15	-	K
Efficiency of working fluid pump	$\eta_{p,\;\mathrm{wf}}$	0.8	-	-
Efficiency of cold seawater pump	$\eta_{p,\;c}$	0.8	-	-
Quantity of condensation sections	n	20	-	-
Fouling resistance of the HTP on working fluid side	$R_{\mathrm{wf}}$	0.7 × 10^−5^	-	$m^{2}\cdot K/W$
Fouling resistance of the HTP on the cold seawater side	$R_{c}$	1.7 × 10^−5^	-	$m^{2}\cdot K/W$
Weighting coefficient	$a_{0}$	0.75	0.45~0.95	-
MFR of cold seawater	${\dot{m}}_{c}$	103.00	93~133	kg/s
Effective volume of the plate condenser	$V_{\mathrm{eff}}$	1.00	0.8~1.2	$m^{3}$
Effective length of the HTP	$L_{\mathrm{eff}}$	1.80	0.75~2.00	m
Width of the HTP	w	1.20	0.75~2.00	m
Effective number of the HTP	$N_{\mathrm{eff}}$	100	70~180	-
Corrugation wavelength of the HTP	Λ	10	7~13	mm
Corrugation angle of the HTP	β	45	30~60	°

## Nomenclature

A	heat transfer area, $m^{2}$
$A_{0}$	heat transfer area of single heat transfer plate, $m^{2}$
$A_{\mathrm{pro}}$	projected area, $m^{2}$
$A_{s}$	cross-sectional area of single flow channel, $m^{2}$
b	plate spacing between two adjacent plates, m
$c_{p}$	specific heat at constant pressure, $J/\left(\mathrm{kg}\cdot K\right)$
$d_{h}$	hydraulic diameter, m
$F_{\mathrm{SP}}$	composite function
f	friction factor
G	mass flow rate per cross-sectional area, $kg/\left(m^{2}\cdot s\right)$
h	enthalpy, J/kg
K	total heat transfer coefficient, $W/\left(m^{2}\cdot K\right)$
L	length, m
m	mass, kg
N	number of heat transfer plates or flow channels
Nu	Nusselt number
n	quantity of condensation sections
P	pumping power, W
p	pressure, Pa
Pr	Prandtl number
Q	quantity of heat transfer, J
R	fouling resistance, $\left(m^{2}\cdot K\right)/W$
Re	Reynolds number
$S_{g}$	entropy generation, J/K
T	temperature, K
t	corrugation pitch, mm
V	volume, $m^{3}$
w	width, m
X	dimensionless corrugation parameter of heat transfer plate
x	vapor quality
Greek letters	
α	surface heat transfer coefficient, $W/\left(m^{2}\cdot K\right)$
β	corrugation angle, ${}^{o}$
δ	thickness, m
ϕ	surface enlargement factor of heat transfer plate
$\eta_{p}$	pump efficiency
λ	thermal conductivity, $W/\left(m\cdot K\right)$
ρ	density, $kg/m^{3}$
μ	dynamic viscosity, Pa⋅s
Λ	corrugation wavelength, mm
ΔT	logarithmic mean temperature difference, K
Δp	pressure drop, Pa
Subscripts	
ave	average value
c	cold seawater
cond	condenser
eq	equivalent value
eff	effective value
i	sequence number of each small condensation section
in	inlet
int	initial value
ios	isolated system
l	saturated liquid state
m	primary minimum value
mm	twice minimum value
opt	primary optimal value
oo	twice optimal value
out	outlet
p	heat transfer plate
sum	total
v	saturated vapor state
wf	working fluid
1, 2, 3,4,5	cyclic state points
Superscript	
⋅	rate, $s^{-1}$

**Abbreviations**

CF	composite function
COR	constructal optimization result
EGR	entropy generation rate
HE	heat exchanger
HRSG	heat recovery steam generator
HTC	heat transfer coefficient
HTP	heat transfer plate
HTR	heat transfer rate
MFR	mass flow rate
OTEC	ocean thermal energy conversion
SLS	saturated liquid state
